# Life philosophy of Bin Kimura

**DOI:** 10.1002/pcn5.145

**Published:** 2023-09-24

**Authors:** Kenjiro Fukao

**Affiliations:** ^1^ Department of Psychology, Faculty of Human Sciences Tezukayama Gakuin University Sakai‐shi Osaka Japan

**Keywords:** *aida*, Bin Kimura, *bios* and *zoé*, life theory, phenomenological–anthropological psychopathology

## Abstract

Bin Kimura, the most internationally renowned Japanese psychopathologist, developed a unique life‐theoretical position in his later years. The concept of “*aida*” or “betweenness,” which was in the social dimension in his earlier thought, came to be called “horizontal betweenness,” and the “vertical betweenness” in the vital dimension came to be emphasized. In relation to his time theory, the “intra festum,” which signifies the tendency to immerse oneself in the present, has come to be highlighted as a direct contact with life. He used many coupled concepts, such as “*mizukara*/*onozukara*” and “reality/actuality,” to contrast his life‐theoretical position with the scientific‐epistemological one. He was also heavily influenced by the ideas of Viktor von Weizsäcker, and superimposed his concept of “vertical betweenness” over Weizsäcker's concept of “ground relationship” by interpreting it as expressing the dependence of individual life (*bios*) on life in general (*zoé*). However, the strongest influences on his life theory were the ideas of Kitaro Nishida, the leading philosopher of the Kyoto School, with whom he had been familiar since his youth. In his last years, Kimura, under Nishida's influence, came to equate life in general with generalized death. Kimura's life philosophy might provide the foundation of psychotherapy by deepening subjective and empathetic understanding of psychiatric patients.

## INTRODUCTION

Bin Kimura (1931–2021) is undoubtedly the most internationally renowned Japanese psychopathologist. Kimura belongs to the second generation of Japanese psychopathologists,^1^ which is also composed of the scintillating personalities of Doi, Kasahara, Yasunaga,^2^ Miyamoto,^3^ and Nakai.^4^ Among them, Kimura was most philosophically oriented and worked most internationally. He was invited to Heidelberg by Tellenbach in 1969 and got acquainted with Blankenburg, resulting in lifelong friendship.

Kimura is regarded as the Japanese representative of the phenomenological–anthropological psychopathology school, but he conducted theoretical explorations with conspicuous originality and philosophical depth that did not fit within that framework. His voluminous psychopathological and philosophical achievements are often divided into three periods. The first period, ranging from the 1960s to the 1970s, is a period of theory of self as represented by his book *Hito to Hito tono Aida* (Between Person and Person) (1972).^5^, ^6^ The second period, ranging from the late 1970s to the 1980s, is a period of theory of time represented by his book *Jikan to Jiko* (Time and Self) (1982).^7^ The third period is a period of theory of life that continues from the late 1980s until the end of his life. This paper outlines Kimura's life theory or life philosophy.

## *AIDA*: HORIZONTAL AND VERTICAL

Among the many concepts that Kimura developed in the process of constructing his own system of theory, “*aida*” is well known. “*Aida*” is an objectified concept of what is usually perceived secondarily as space between existing individuals, and in English it should be described as “betweenness,” but Kimura says that “*aida*” exists not secondarily but primarily. He writes

Just when the self encounters the non‐self, like a spark flying out, the self and the non‐self arise from *something*. …the individual is what has separated from this *something* by the encounter between the self and the non‐self. This *something* exists before the individual.^5^, ^6^

That “something” is “*aida*” or “betweenness,” and it is not that “betweenness” arises between individuals, but on the contrary, individuals arise on both sides of “betweenness.”

In his early book, *Between Person and Person* (1972), Kimura was influenced by the theory of “*Aida‐gara‐teki Sonzai*” (間柄的存在: interpersonal existence) by the Japanese philosopher Tetsuro Watsuji, and to that extent, “*aida*” meant the horizontal relationship between people living in each society. However, in his later book, *Aida* (1988), which heralds the beginning of the third period, the concept of “*aida*” belongs to a completely different dimension from such a social one. The “Introduction” to *Aida* states the hypothesis that is the premise of the entire book. It says, “There is what is to be called the ground of life in general on this earth, and the fact that each of us is alive means that our existence maintains a connection with this ground of life in general, both in action and sensation.”^8^, ^9^ In this vital dimension, “*aida*” or “betweenness” came to be seen as something that connects individual living beings, or rather, as something that fundamentally supports all living beings.

Kimura explains this theory with the following metaphor:

Imagine a fountain that flows from a source full of vital water pressure through the outlet of an individually separated physical being (called ‘mi’ or body), and the curve of the water, which arcs differently according to the characteristics of each vent, is the individual self.^8^, ^9^

Namely, Kimura believes that although “*aida*” may appear to exist on the level of social human relations on the surface, it essentially derives from the fundamental spontaneity shared by all living beings, not just all humans. Then the concept of “*aida*” is supposed to be doubly structured, that is, the “horizontal *aida*” existing between individual living beings, and the “vertical *aida*” existing between each living being and the fundamental spontaneity or life in general.

Kimura also describes this “vertical *aida*” as “life‐theoretical difference,” reinterpreting “ontological difference” in Heidegger's philosophy from a life‐theoretical standpoint. While “ontological difference” means the dimensional difference between individual beings and existence, his “life‐theoretical difference” means the dimensional difference between individual living beings and life in general. The peculiarity of the concept of “*aida*” lies in stressing that human relationships in everyday life (horizontal *aida*) directly communicate with the metaphysical “life‐theoretical difference” (vertical *aida*).

## TO THE DEPTH OF THE PRESENT

Kimura's second period of work revolved around the theory of time or temporality. In his theory called the “Festum Theory” that corresponds the three aspects of time to the three major mental illnesses from the standpoint of the anthropological school, he regarded various mental illnesses as the extreme of the possibilities of the human mind. Namely, depression is a disease of the past‐oriented posture or “post festum,” schizophrenia is one of the future‐oriented posture or “ante festum,” and mania and epilepsy are of the present‐oriented posture or “intra festum.”^7^

The focus of the transition from time theory to life theory in the third period was “intra festum.” In the first place, “post festum” is an idiom, and “ante festum” is a word borrowed from Marxist thinkers Georg Lukács and Josef Gabel, while “intra festum” is a word created by Kimura. In contrast to “post festum,” which is the mentality of nostalgia and regret for the festival that has already passed, and conversely, “ante festum,” which is the mentality that trembles with anticipation for the coming festival, “intra festum” means the enthusiastic and immersed mentality in the midst of the festival.

While “post festum” and “ante festum” are qualitative anomalies in the inclination of temporality toward the past and future, respectively, “intra festum” is a quantitative anomaly of excessive adhesion to the present. In epileptic seizures and manic agitation, patients show excessive immersion in the present, and its connections with the past and the future are broken, resulting in loss of consciousness and disruption of behavior.

The fanatical and immersive mentality of the “intra festum” is an undivided state of self and others in which the whole group or life in general and the individual ego are not distinguished. Thus, “intra festum” is thought to be a state in which the individual mind is in direct contact with the life in general that sustains all living beings.

## “MIZUKARA” AND “ONOZUKARA”

Kimura uses musical ensembles as an example to illustrate the concept of “*aida*” (he was also an excellent piano performer).^8^, ^9^ During an ensemble, the individual players play their instruments while listening to the music played by everyone, but if you are too particular about your own will, you shall disturb the overall performance, and conversely, if you adjust too much to the whole, your own performance will be lackluster. Music is ideally established when one's will and conformity to the whole are harmonized and united. At such times, it becomes impossible to distinguish whether it is your will or everyone's will that controls your performance. That state is the state in which “*aida*” or “betweenness” reigns.

Kimura explains this dual structure of human agency by contrasting the two Japanese words “*mizukara*” and “*onozukara*.”^8^, ^9^, ^10^ “*Mizukara*” is a word that expresses the free will of the individual, and “*onozukara*” is a word that expresses that things will happen naturally without anyone's will, but these two words are expressed by the same kanji character “自.” And this character originally represents “beginning” or “origin.” Namely, in the Eastern perception of causality, the free will of the individual and the natural process are not opposed as in the modern Western perception of causality, but rather superimposed and united. From there, Kimura thought that the disturbed harmony between “*mizukara*” and “*onozukara*” was the essence of mental illnesses, especially schizophrenia. Patients with schizophrenia are thought to be controlled by “*mizukara*” without harmony with “*onozukara*,” as reflected by their awkwardness in social interaction.

## METANOESIS

Kimura coined the term “metanoesis” to describe the super‐individual action that moves an individual, which is expressed by “*onozukara*.”^8^, ^9^ This concept was devised based on the coupled concepts of “noema/noesis” derived from Husserl's phenomenology. Noema is the object of action of consciousness and noesis is the action of consciousness that forms noema, while “metanoesis” is the action before consciousness that forms noesis. Applying this to the example of an ensemble, the music played is noema, the will of each player is noesis, and the action of regulating the will of each player to bring the music together as a whole is “metanoesis.”

Kimura also explains “metanoesis” using the example of a flock of migratory birds.^11^, ^12^ In such kind of birds in which a large number of individuals gather and migrate in groups, the individual subjectivity of each bird must be consistent with the subjectivity of the entire flock, which Kimura calls “group subjectivity” (集団主体性), or it will not be possible to succeed in long‐distance migration. Human beings are also inherently social animals and at the bottom of the individual subjectivity that is always conscious exists group subjectivity that is not always conscious. If the relationship between individual subjectivity and group subjectivity is broken, normal life cannot be performed.

Kimura interprets ego disorder, the central symptom of schizophrenia in which the distinction between self and others is blurred, as a disharmony between individual subjectivity and group subjectivity.^12^ In schizophrenia, individual and group subjectivity, which should be in harmony in humans as social animals, are separated, and because “*mizukara*” and “*onozukara*” diverge, it is felt as if what is happening inside of oneself is happening outside, and conversely, what is happening outside is happening inside.

## REALITY AND ACTUALITY

By making full use of various contrasted concepts, Kimura tried to bring his own life‐theoretical position to light in contrast to cognitive and linguistic position. One of them is the contrast between the verbs “*aru*” and “*iru*,” which mean “presence” in Japanese.^9^ Both correspond to the verb “be” in Western languages, but “*aru*” means “objective, cognitive, and contingent existence,” while “*iru*” means “subjective, actional, and necessary existence.” Kimura linked this contrast to Nietzsche's concept “will to power” (*Wille zur Macht*).^13^, ^14^ That is, Nietzsche's statement that “the will to power imprints the character of existence on generation” is interpreted as that an individual existence is made by limiting “*iru*” that is necessary as an action of life in general into the contingent “*aru*.” In this way, from a life‐theoretical standpoint, Kimura regards “truths” that exist in the way of “*aru*” to be fabricated fictions as objects of recognition.

In relation to this, Kimura takes a cue from Deleuze's interpretation of Bergson's philosophy and uses the dual concept of “reality/actuality.”^15^ Reality is the world of objective things that exist in the public realm, whereas actuality is the world of subjective actions and intuition that exist in the private realm. According to Kimura, in depersonalization, reality as the object of recognition has not been lost, but actuality as the vital feeling has been lost. This actuality is fundamentally based on a connection to life in general, which is described as virtuality or the matrix of actuality.

As can be easily seen, “*aru*” corresponds to reality and “*iru*” to actuality. Moreover, this contrast is also valid for the difference between science and clinical position. In other words, by taking the standpoint of actuality, Kimura differentiated himself from scientific psychiatry, which is bound by reality or truth.

## GROUND RELATIONSHIP

Kimura in the third period was greatly influenced by the ideas of Viktor von Weizsäcker, who was a neurologist and medical philosopher and proposed “medical anthropology” (*medizinische Anthropologie*). In his 40s he had translated Weizsäcker's main work *Der Gestaltkreis* into Japanese with his colleague Toshihiko Hamanaka, but after his 60s he translated other works—“*Studien zur Pathogenese*,” “*Der Kranke Mensch*,” “*Gestalt und Zeit*,” “*Anonyma*,” and “*Pathosophie*”—one after another, deepening his understanding of Weizsäcker's thoughts.

Kimura repeatedly cites the following three quotes from Weizsäcker's texts.^16^

The first is “In order to study living beings, one must relate oneself to life” (*Um Lebendes zu erforschen, muß man sich am Leben beteiligen*). In other words, medicine that deals with human beings, who are living organisms, must be related to life in general.

The second is that “Life itself never dies, only individual living beings die” (*Aber das Leben selbst stirbt nicht; nur die einzelnen Lebewesen sterben*). That is, individual living beings die, but life in general, to which they all belong together, does not die.

The third is “Life enters life through physicality” (*mit der Leiblichkeit kommt Leben ins Leben*). That is, life in general becomes a real‐life activity through the bodies of individual living beings.

The main concepts that Kimura drew from Weizsäcker's thoughts are the concept of the subject (*Subjekt*) as the generative and annihilating boundary between organisms and the surrounding world (*Umwelt*), and the concept of “ground relationship” (*Grundverhältnis*) that individual living beings have with respect to life in general. The former corresponds to the “horizontal *aida*” that gives rise to the individual ego, and the latter corresponds to the “vertical *aida*” that forms the basis of the individual ego. In this way, Kimura interpreted the life‐theoretical aspects of Weizsäcker's thoughts and extracted concepts that supported his theory of “*aida*.”

Borrowing the concepts of classical philologist Karl Kerényi, Kimura in his later period called individual life “*bios*” and life in general “*zoé*.”^17^ He explained that Weizsäcker's concept of “ground relationship” refers to the *bios*'s dependence on *zoé*.

## DEATH AS LIFE

The greatest influence on Kimura's thought during his lifetime was that of Kitaro Nishida, the founder of the Kyoto School of philosophy and a pioneer of modern Japanese philosophy.^18^ Regarding the relationship between the self and others, Nishida argues that the self and others do not meet directly, but that there is an “absolute other” at the bottom of the self, and there is an “absolute other” at the bottom of the other, and that they meet each other there (“I and Thou”). Kimura adopted this theory, superimposing Nishida's “absolute other” with Weizsäcker's “ground of life.”^19^

In his later years, Kimura gradually began to discuss the issue of death, perhaps because he was conscious of his own lifespan.^20^, ^21^ He appreciated Freud's concept of “death drive,” but argued that Freud was wrong in thinking that what the “death drive” was aiming for was the death of the individual. This is because, for Kimura, the “desire to die” was not the impulse of individual life to return to inorganic matter as Freud thought, but rather the impulse to return to life in general or *zoé*.

Nishida's “absolute other” was a medium for self and others to communicate, but for Kimura, it was not only a medium that connected individual lives, but also the matrix to which individual life returns when it dies. In this sense, Kimura equated life in general as “absolute other” with generalized death. Kimura's theory of self in his first period of thought incorporates Nishida's concept of “absolutely paradoxical identity” (絶対矛盾的自己同一), in which the absolute distinction between oneself and others makes absolute identity of oneself and others possible, using the concept of “death as life” (死即生) figuratively. But, in his third period of thought, “death as life” is understood as literally meaning “life = death.”

## SIGNIFICANCE OF KIMURA'S LIFE PHILOSOPHY

What is the significance of Kimura's life philosophy as we have seen above for psychopathology?

One of the pioneers who developed a life‐theoretical position in psychopathology is Eugène Minkowski, who, under the influence of Bergson's life philosophy, characterized schizophrenia as “a lack of vital contact with reality.” In fact, Kimura was influenced by Minkowski's theory through his mentor Masashi Murakami, who was the Japanese translator of “Minkowski's book *Schizophrenia*.” However, the position of life in Minkowski and that of Kimura are quite different. While Minkowski focused only on individual life, for Kimura life exists *between* individual living beings (horizontal *aida*), which also means it exists *between* individual living beings and life in general (vertical *aida*), so that individual life could not exist without the connection with life in general.

On the surface, Kimura's theory of “*aida*” looks like a theory of sociality. However, as explained above, for Kimura, “*aida*” is not based on social recognition as a higher‐order function peculiar to humans, but on the connection with life in general, which is inherent in even the most primitive creatures. It is because of this life‐theoretical belief that although Kimura sympathized with the anti‐psychiatry movement, he did not support cultural relativism or social constructionism.^22^

The present setting of psychiatric practice, which is guided by operational diagnostic systems and algorithmic treatments, appears as if it did not need the human relationship between the patient and the therapist. All clinicians know, nevertheless, that psychiatric practice needs it as before. However, it is hard to establish the human relationship with patients who have trouble in sociality, like autistic or delusional schizophrenic patients, on the level of verbal exchange. The life‐theoretical perspective that Kimura advocated may effectively help in this situation because it opens the deeper level that the patient and the therapist can communicate each other nonverbally, both as living beings.

## AUTHOR CONTRIBUTIONS

Kenjiro Fukao contributed solely the whole work.

## CONFLICT OF INTEREST STATEMENT

The author declares no conflicts of interest.

## ETHICS APPROVAL STATEMENT

N/A

## PATIENT CONSENT STATEMENT

N/A

## CLINICAL TRIAL REGISTRATION

N/A

## Data Availability

N/A
